# The Healthy Democracy Kit: design, implementation, uptake, and impact of a novel voter registration toolkit for healthcare settings

**DOI:** 10.1186/s12889-023-15800-x

**Published:** 2023-05-26

**Authors:** Madeline M. Grade, Alexander W. T. Reardon, Yoonhee P. Ha, Adi Steinhart, Alister F. Martin

**Affiliations:** 1 Department of Emergency Medicine, University of California San Francisco, San Francisco, USA; 2 Department of Emergency Medicine, The McGaw Medical Center of Northwestern University, Chicago, USA; 3 Perelman School of Medicine, Center for Health Equity Advancement, and Center for Health Incentives & Behavioral Economics, University of Pennsylvania, Philadelphia, USA; 4 School of Medicine, New York Medical College, Valhalla, USA; 5 Department of Emergency Medicine, Massachusetts General Hospital, Boston, USA

**Keywords:** Civic health, Voter registration, Health equity, Behavioral insights

## Abstract

**Background:**

Access to voting is increasingly recognized as a social determinant of health. Health equity could be improved if healthcare workers (HCWs) routinely assessed the voter registration status of patients during clinical encounters and helped direct them towards appropriate resources. However, little consensus exists on how to achieve these tasks efficiently and effectively in healthcare settings. Intuitive and scalable tools that minimize workflow disruptions are needed. The Healthy Democracy Kit (HDK) is a novel voter registration toolkit for healthcare settings, featuring a wearable badge and posters that display quick response (QR) and text codes directing patients to an online hub for voter registration and mail-in ballot requests. The objective of this study was to assess national uptake and impact of the HDK prior to the 2020 United States (US) elections.

**Methods:**

Between 19 May and 3 November 2020, HCWs and institutions could order and use HDKs to help direct patients to resources, free of cost. A descriptive analysis was conducted to summarize the characteristics of participating HCWs and institutions as well as the resultant total persons helped prepare to vote.

**Results:**

During the study period, 13,192 HCWs (including 7,554 physicians, 2,209 medical students, and 983 nurses) from 2,407 affiliated institutions across the US ordered 24,031 individual HDKs. Representatives from 604 institutions (including 269 academic medical centers, 111 medical schools, and 141 Federally Qualified Health Centers) ordered 960 institutional HDKs. Collectively, HCWs and institutions from all 50 US states and the District of Columbia used HDKs to help initiate 27,317 voter registrations and 17,216 mail-in ballot requests.

**Conclusions:**

A novel voter registration toolkit had widespread organic uptake and enabled HCWs and institutions to successfully conduct point-of-care civic health advocacy during clinical encounters. This methodology holds promise for future implementation of other types of public health initiatives. Further study is needed to assess downstream voting behaviors from healthcare-based voter registration.

## Introduction

Civic participation, which includes voting in elections, is associated with better self-reported physical and mental health. This is described across a growing body of cross-sectional and longitudinal studies, and the mechanism seems to be multifactorial and bidirectional [1]. Many authors hypothesize that some individual health benefits are mediated via increases in social capital. On a larger scale, greater participation in the decisions of a representative government may yield policies more aligned with one’s individual interests, many of which (e.g., laws regarding health access, employment, housing) can directly impact downstream health [2].

Conversely, when marginalized communities abstain from voting–whether due to alienation, suppressive policies, or excess mortality–it can negatively affect their health [3–5]. The political exclusion of racial and ethnic minorities, individuals with disabilities, and low-income groups can skew subsequent public policy away from their interests. In turn, this can negatively impact the social determinants of health (SDOH) for these already marginalized groups. Further exacerbating this cycle is the negative impact of poor health itself on rates of voter turnout. Thus, political disempowerment is associated with poorer health and poor health with further political disempowerment [2, 6]. In the United States (US), there is significant overlap between the groups most affected by inequities in health and the groups disproportionately disenfranchised by suppressive voting policies, namely people with less education, people with lower income, and racial and ethnic minority groups [7]. Given this context, large-scale “civic health” initiatives aimed at addressing disparities in voting access may in turn provide a direct means of addressing health inequities for marginalized communities.

In June 2022, the American Medical Association recognized access to voting as a SDOH [8]. Healthcare workers (HCWs) and institutions can play an important role in addressing this social determinant by promoting the civic participation of the communities they serve. The first step to doing so is to expand voter registration access, particularly amongst historically disenfranchised populations. The 1993 National Voter Registration Act allows for voter registration at healthcare sites providing “public assistance”, including treating patients with government-funded insurance plans [9].

Expanding voter registration access through healthcare settings is not without precedent. Several individual clinics have described successful voter registration initiatives at the local level [10–12]. Additionally, given the significant overlap between voters marginalized by the political and healthcare systems, the National Association of Community Health Centers (NACHC) created an implementation guide for clinic-based voter registration and mobilized over 200 community health centers to participate in 2012 [13, 14]. However, prior to 2020, there was no readily available toolkit designed to mobilize HCWs and institutions of all types to expand voter registration access in healthcare settings on a nationwide scale.

Vot-ER (vot-er.org), a nonpartisan 501(c)(3) nonprofit, was founded with the vision of addressing this need. Furthermore, as the coronavirus disease 2019 (COVID-19) pandemic led to restrictions on in-person visits to traditional voter registration sites like departments of motor vehicles and made in-person voting a public health risk, the organization recognized the importance of identifying new channels for promoting voter registration and voting by mail. To that end, a diverse team including designers, HCWs, students, and volunteers developed and distributed the Healthy Democracy Kit (HDK), a readily scalable toolkit that HCWs and institutions could use to connect patients with voting resources during clinical encounters. The purpose of this study was to describe the design, implementation, uptake, and impact of this initiative ahead of the November 2020 US elections.

## Methods

### Design

#### Healthy Democracy Kits

**Fig. 1:**
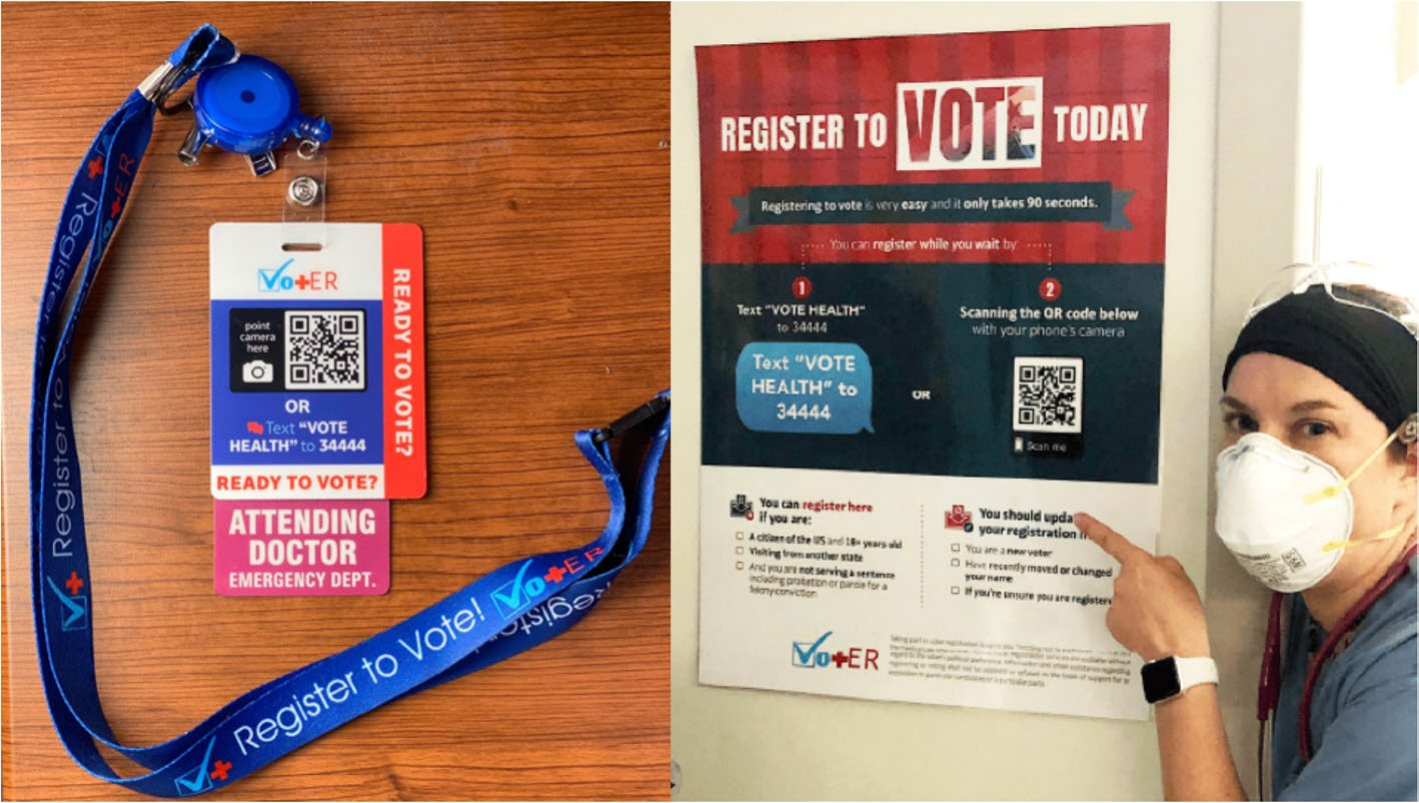
Photos of key Healthy Democracy Kit components, including examples of a badge and lanyard (left, photo courtesy of Dr. Alister Martin) and a poster (right, photo courtesy of Dr. Ashlee Murray)

Vot-ER developed two types of HDKs: individual and institutional (Fig. 1). The individual HDK consisted of a lanyard and a plastic badge that could also attach directly to a badge reel. Institutional HDKs included badges and posters for clinical settings, provided as digital files that could be printed and distributed locally. All badges featured a URL for an online instructional video for HCWs. As the initiative progressed, institutional HDKs grew to include additional digital tools such as smartphone screensavers and video conferencing backgrounds (Appendix 1).

**Fig. 2:**
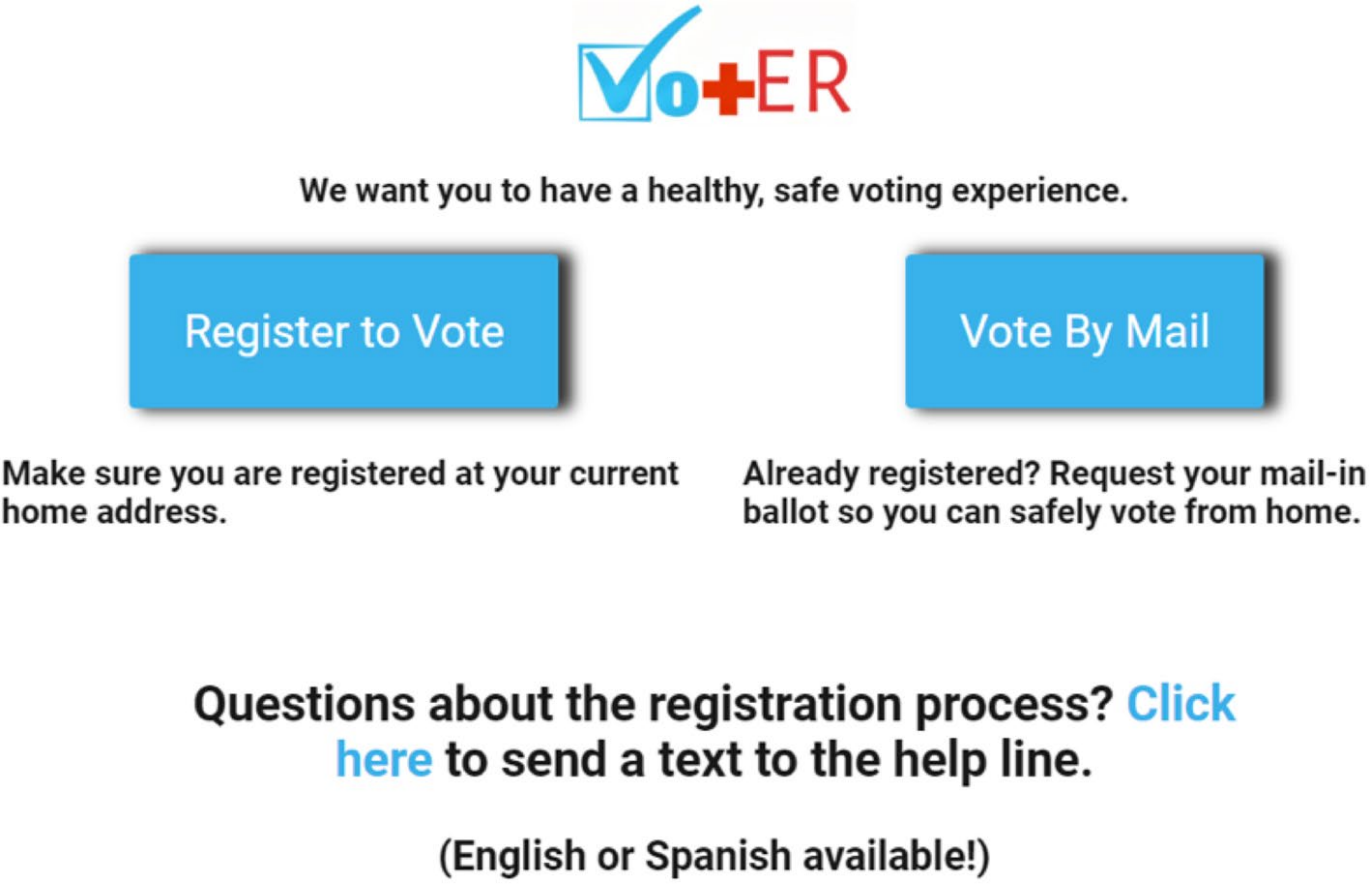
The landing page for the HDK online hub, featuring links for voter registration, mail-in ballot request, and a bilingual help line in one place

The HDK was designed to maximize HCW workflow efficiency and simplify the patient experience. The key functional elements of HDKs were quick response (QR) and text codes located on the badges and posters. Once participants scanned the QR code or texted “VOTE HEALTH” to “34444” with their smartphone, they were automatically directed to an online hub (Fig. 2) containing links for 1) a third-party voter registration website (turbovote.org), 2) a third-party mail-in ballot request website (vote.org), and 3) a 24-h mobile helpline in English and Spanish.

HCWs using individual HDKs were instructed to provide a neutral prompt about voter registration during clinical encounters (e.g., “Do you have a plan to vote safely in the upcoming election? If not, here are some resources.”), at which point they could display their badges for patients to scan at the point-of-care. Institutions that displayed codes on posters in waiting areas, after-visit summaries, or other site-based materials created opportunities for passive prompting of patients. HCWs and institutions did not conduct voter registration directly; by design, patients completed the next steps of inputting their information themselves on their smartphones, allowing HCWs to move forward in their clinical workflows.

#### Customization

We provided institutions with the option of customizing their HDKs with a text code of their choosing (e.g., “VOTE PENN”, “VOTE KIDS”) and a unique trackable QR code. Customization allowed for near real-time tallying of voter registration and mail-in ballot requests initiated by each institution (see *Impact*).

### Implementation

#### Promotion

**Fig. 3:**
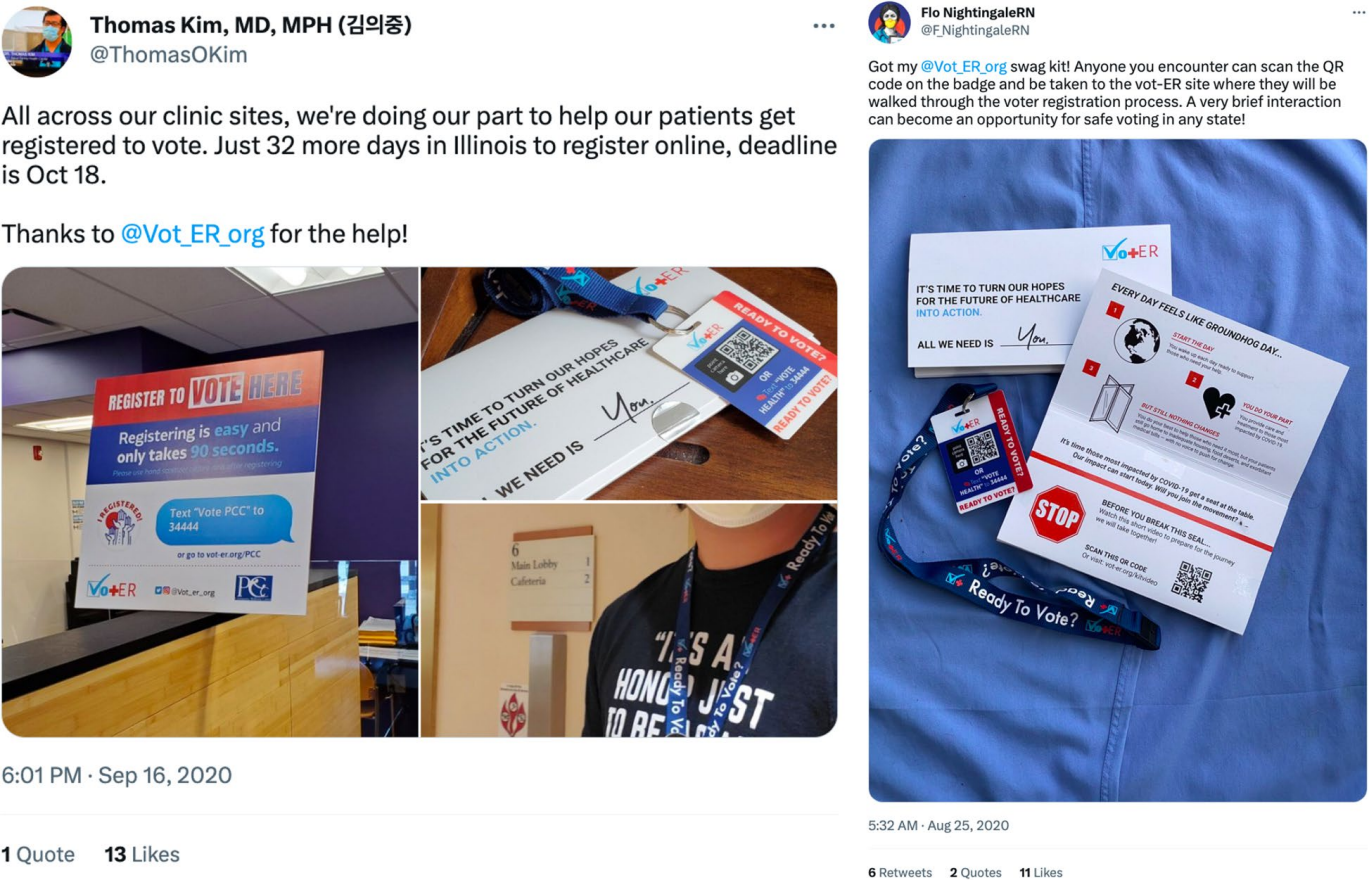
Examples of social media posts about the Vot-ER initiative. A physician shared how his clinic sites were using Healthy Democracy Kits (left) [14]. A registered nurse shared their generic badge next to the informational folder it was mailed in on Twitter (right) [15]

Vot-ER promoted the HDK via informational webinars, professional organization listservs, and social media. Many participants shared photos of themselves using HDKs on their social media accounts, which helped generate and propagate interest across peer networks (Fig. 3) [15, 16].

#### Orders and data collection

Individual HCWs ordered HDKs by completing a brief Vot-ER website form, with input fields for contact information, affiliated institution and state, professional role, and number of toolkits requested. Institutions completed a similar form with an additional input field for optional kit customization specifications. No data were collected on the subsequent HCW users of institutional HDKs.

#### Funding

Massachusetts General Hospital (MGH) and ideas42 funded the production and shipping of physical HDKs, which were provided to HCWs at no cost. Volunteers assembled the physical HDKs for shipping. Institutions received digital HDKs for free and covered their own costs of producing badges and posters locally.

### Measurement

#### HCW and institutional uptake

We conducted a descriptive analysis of individual and institutional HDK orders placed between 19 May and 3 November 2020. A subset of this data has been described previously in analyses of pediatric [17] and medical school [18] initiatives. Institutions were defined as academic if they sponsored a residency program or had a major affiliation with a medical school in the American Medical Association’s Residency and Fellowship Database [19]. We identified Federally Qualified Health Centers (FQHCs) using the national FQHC Database [20] and accredited allopathic and osteopathic medical schools using member lists from the Association of American Medical Colleges and American Association of Colleges of Osteopathic Medicine [21, 22].

To better understand the patient populations served by HCWs and institutions that used HDKs, we linked affiliated institution and institution ZIP codes to publicly available datasets with ZIP code-level socio-demographic characteristics, including the 2013 Urban Influence Codes [23] (metropolitan versus non-metropolitan), Census Bureau’s 2019 American Communities Survey accessed via the R package “tidycensus” [24] (percent non-white population and average household income), and the 2018 National Neighborhood Data Archive [25] (percent registered voters).

#### Impact

Non-customized HDKs all shared a generic text code and corresponding QR code that were both connected to a single online hub, whereas each customized HDK linked to a unique hub. For each code, we utilized Google Analytics data to measure the number of people who initiated voter registration and mail-in ballot requests (i.e., clicked the corresponding “Register to Vote” and “Vote by Mail” links to our third-party partner websites). Subsequent data entered into the third-party websites, including completed registrations, were not accessible by the study team or included in this study, but a third-party overview of key demographics and aggregate voter turnout for participants is referenced in the Discussion.

For institutions utilizing customized codes, we created an online leaderboard that ranked institutions by the number of people they helped initiate voter registration or mail-in ballots. This tool provided feedback by automatically updating every 15 min and allowed institutions to monitor their own performance and make peer comparisons, such as in our previously described voter registration competition amongst 80 US medical schools [18].

### Statistical analysis

Descriptive statistics were derived using RStudio [26].

### STROBE Guidelines

We adhered to the Strengthening the Reporting of Observational Studies in Epidemiology (STROBE) guidelines [27].

## Key results

### Uptake

#### Individual HDK orders

Between 19 May and 3 November 2020, 13,192 HCWs ordered 24,031 individual HDKs. The largest increase in individual HDK orders occurred during the month of August 2020, which coincided with the inaugural Civic Health Month (civichealthmonth.org), when over 100 civic engagement and healthcare partners promoted the importance of civic health across their institutions [28]. From 1 to 31 August, the cumulative total of individual HDKs ordered increased by 119% from 3,838 to 8,402.

**Table 1:** Characteristics of Individual HDK Users

Individual HDK Orders (*n* = 13,192)	
**Professional Role**	
Physician	7,554 (57%)
Medical Student	2,209 (17%)
Nurse	983 (7.5%)
Social Worker	637 (4.8%)
Advanced Practice Provider	472 (3.6%)
Other	1,057 (8.0%)
Not Answered	280 (2.1%)
**Affiliated Institution Type**	
Academic Medicine	9,608 (73%)
Outpatient or Community-Based	1,928 (15%)
Hospital-Affiliated	10,492 (80%)
Pediatric Only	1,482 (11%)
Federally Qualified Health Center	756 (5.7%)
Other	280 (2.1%)
Institution Not Specified	554 (4.2%)

Most individual HDK users identified as physicians (7,554, 57%), many of whom reported their experience level (4,590 attendings, 246 fellows, and 1,985 residents) (Table 1). Other professional roles with high participation included medical students, nurses, and social workers. In total, HCWs who ordered HDKs spanned over 50 distinct professional roles including administrative staff, therapists, technologists, interpreters, and chaplains (Appendix 2).

**Table 2:** Characteristics of Institutions with Individual HDK Users or Institutional HDKs

	**Institutions of HCWs with Individual HDKs** **(** ***n*** ** = 2,407)**	**Institutions with Institutional HDKs** **(** ***n*** ** = 604)**
**Institution Characteristics**		
Academic Medicine	665 (28%)	269 (45%)
Medical School	175 (7.3%)	111 (18%)
Clinic or Community-Based	1,170 (49%)	276 (46%)
Hospital or Mixed Setting	1,082 (45%)	303 (50%)
Pediatric-Only	301 (13%)	100 (17%)
Federally Qualified Health Center	211 (8.8%)	141 (23%)
Other (e.g., Nonprofit, Industry)	200 (8.3%)	34 (6%)
**Institution ZIP Code Characteristics**		
Metropolitan Area	2,265 (94%)	566 (94%)
Average Percent Non-White	34%	38%
Above-Average Percent Non-White	1,770 (74%)	471 (78%)
Average Household Income	$67,748	$62,349
Below-Average Household Income	1,192 (50%)	345 (57%)
Average Percent Registered to Vote	90%	88%

**Fig. 4:**
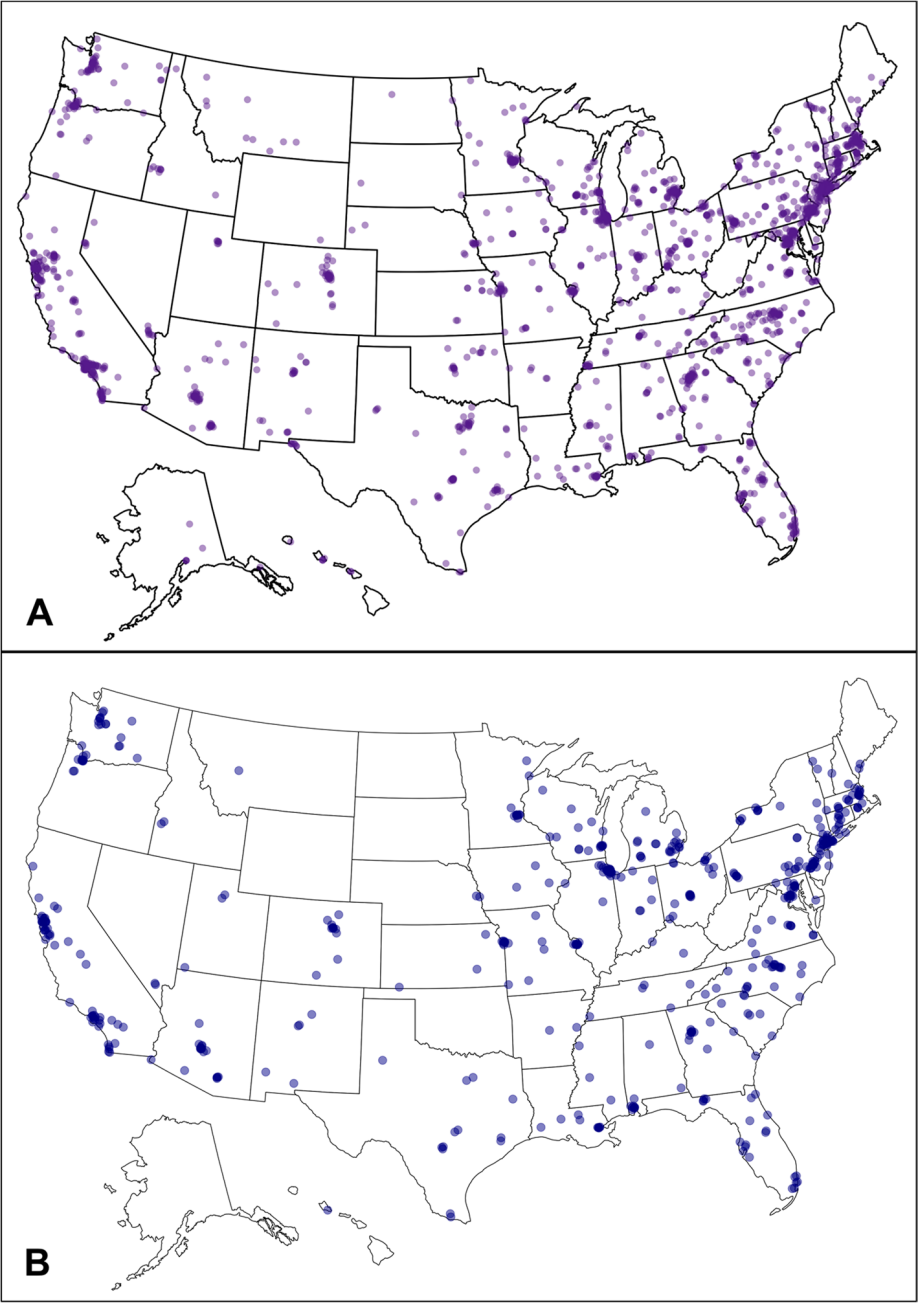
Two maps of the United States illustrating the ZIP codes of the affiliated institutions of (**A**) the healthcare workers that ordered individual Healthy Democracy Kits (HDKs) and (**B**) the institutions that ordered institutional HDKs. More than one individual, department, or division may have ordered toolkits from a given institution. The map marker for a given ZIP code may correspond to one or more institutions. Created in Rstudio [26].

HCWs were affiliated with institutions from all 50 states and the District of Columbia (Fig. 4). The states with the greatest number of individual HDK orders were California (1,416, 11%), Pennsylvania (1,250, 9.5%), and New York (1,087, 8.2%). The majority of HCWs were affiliated with an academic medical center (Table 1). More than 10% of HCWs were affiliated with a pediatric-only hospital or clinic. HCWs were affiliated with 2,407 distinct institutions, including a mix of hospitals, medical schools, freestanding clinics (including over 200 FQHCs), and other community-based organizations (Table 2). Affiliated institutions were overwhelmingly located in metropolitan areas with a greater proportion of non-white residents than the national average.

#### Institutional HDK orders

During the initiative, 604 unique institutions (Table 2) from 43 states and the District of Columbia placed 960 institutional HDK orders. The number of orders per institution ranged from 1 to 10. The number of orders exceeded the number of institutions because multiple departments or divisions within the same institution could request a toolkit. The number of toolkits requested ranged from 1 to 10,000, with a median of 50 [Q1 25, Q3 100]. Approximately 519 (54%) institutional HDK orders were customized.

Compared to affiliated institutions for HCWs that placed an individual order, institutions that submitted institutional HDK orders were more commonly academic medical centers, FQHCs, or pediatric-only institutions (Table 2). The majority of affiliated ZIP codes corresponded to metropolitan areas and had a greater than average percentage of non-white residents.

### Impact

Between 19 May and 3 November 2020, HCWs and institutions used HDKs to help initiate 27,317 voter registrations and 17,216 mail-in ballot requests in healthcare settings. There were an additional 2,179 click-throughs to other pre-election resources such as polling location lookup and vote-tripling information. Custom QR or text codes were utilized in 12,440 (46%) initiated voter registrations and 9,248 (54%) initiated ballot requests. Institutions that successfully implemented custom codes initiated a median of 316 [197, 446] registrations and 167 [111, 262] mail-in ballot requests. The institution with the greatest number of people helped was Penn Medicine, with 1,692 initiated registrations and 2,070 initiated ballot requests.

## Discussion

This study describes the largest healthcare-based voter registration initiative to date, with unprecedented reach in terms of numbers and types of participating HCWs and institutions, geographic spread, and people helped. This initiative had several unique strengths that facilitated rapid adoption and utilization nationwide.

One major innovation was the technologic approach, which centered around QR and text codes and leveraged increasing rates of smartphone ownership (over 85% of Americans) and online voter registration [29, 30]. This digital method enabled efficient interactions between HCWs and patients, promoted patient-directed education and follow-through, enabled tracking and gamification, and was easy to implement and scale across many types of healthcare settings and geographic locations. Additionally, the use of professional and social networks enabled rapid and organic uptake in just a few months. Several additional strengths stem from the behavioral insights-informed approach used in designing and implementing the HDK. These included the concepts of simplicity, visual saliency, feedback, peer comparisons, gamification, and sustainability, which have been shown to increase many behaviors [31].

**Simplicity:** The HDK was designed to be intuitive and low-effort for HCWs and institutions to acquire, implement, and scale. It was offered at no cost and designed to minimize workflow disruption. Additionally, once a badge was donned, it naturally became part of a HCW’s uniform and eliminated the need to remember to carry around materials. The single-hub website design and helpline streamlined resource access for patients.

**Visual saliency:** Badges were designed to be more colorful and larger than hospital IDs, with an exposed banner asking, “READY TO VOTE?”. This visual nudge helped capture the attention of patients and HCWs alike, facilitating natural discussions about voter registration with patients and prompting interested colleagues to obtain their own HDKs.

**Feedback, peer comparisons, and gamification:** The online leaderboard provided participants with regular feedback in near real time. Participants may have been motivated by overall state or peer institution comparisons, as demonstrated in the highly effective medical school competition [18]. Recently, Vot-ER began offering individual HCWs the option to request custom trackable badges independent of their institutions, allowing participants to receive feedback on their individual impact (vot-er.org/track-your-impact).

**Sustainability:** All QR and text codes will remain active year-round and through multiple election cycles. HDK users continue to receive regular communications and opportunities to learn and connect.

This initiative and analysis had several limitations. First, tools were not directly usable for individuals lacking a smartphone nor individuals living in states without online voter registration. HCWs and institutions may address this first issue by setting up digital kiosks in waiting areas (Appendix 3) [32], lending patients hospital-owned tablets, or keeping paper registration forms on hand. Second, certain types of HCWs and institutions were overrepresented. The majority of individual participants were physicians and medical students affiliated with urban academic institutions (perhaps because of promotion via professional networks and social media), whereas institutional participants were more likely non-academic, FQHC, or pediatric-only (perhaps influenced by endorsement from the NACHC and American Academy of Pediatrics). Third, the data collected and used in this study were limited. HDK order forms did not collect comprehensive demographic data on individuals or institutions nor survey participants about their motivations. Socio-demographic characteristics of institution ZIP codes exclude patients who do not reside near their respective healthcare institutions. The number of click-throughs via our online hubs, while offering some insight into the number of conversations about voting in clinical settings, does not equate to (and likely overestimates) completed voter registrations, mail-in ballot requests, and/or subsequent ballots cast. However, it is important to note that many people have never been asked about their voter registration status [33]. Even a first inquiry about registration status or a click-through to helpful resources, especially when prompted by a trusted messenger such as a HCW or institution [34], may serve as an effective first step towards empowering patient voices, particularly amongst historically disenfranchised populations.

Overall, HDKs offer a novel and effective approach to engaging HCWs in advocacy at the point-of-care. No prior initiative describes a trackable code-based badge worn by HCWs, a tool which could be easily implemented for other types of advocacy campaigns. Compared to prior work, this initiative mobilized an unprecedented number and breadth of HCWs across the country to help as many as 46,712 people prepare to vote in 2020 [35]. Furthermore, this initiative overcame workflow barriers, normalized conversations about voter registration in healthcare settings, and demonstrated scalability and sustainability.

The degree and rapidity of uptake signals a high level of enthusiasm amongst participating HCWs during 2020. In fact, US physicians, perhaps spurred by issues such as the COVID-19 pandemic and protests of racial inequities, were shown to have a higher voter turnout than the general population in 2020, in contrast to prior elections [36]. We believe this is partly reflective of a culture shift in medicine towards 1) viewing civic participation as a SDOH and taking steps to address disparities in voting access in clinical settings [8], and 2) participating more visibly in advocacy around important healthcare issues [37]. This is underscored by the involvement of thousands of health professions students in our initiative and recent calls from medical students to include civic health and advocacy training in medical curricula [37, 38]. To date, HCWs across the country continue to order HDKs, and we anticipate that similar healthcare-based voter registration initiatives will continue to grow.

The most exciting impact of this work is its potential to directly improve the SDOH of access to voting, and in turn to affect downstream inequities in civic representation and health outcomes. Promisingly, an initial third-party analysis by TurboVote suggests that our inaugural initiative was indeed effective in starting to address disparities in voter registration and in driving subsequent voter turnout. TurboVote reported that 84% of individuals helped by Vot-ER’s toolkit in 2020 completed voter registration and 85% of these registrants subsequently voted in the general election [39]. Of these voters, 36% were people of color (compared to 26% in the general electorate) and 56% were under 35 years old (compared to 24%) of the general electorate. They also noted that voters who were expected to be “low propensity” turned out to vote 20% more than their counterparts in the general electorate. While detailed analyses of registration and turnout analysis were outside the scope of this study and are forthcoming, these initial results seem to indicate successful empowerment of underrepresented groups in the 2020 election as a direct result of our healthcare-based initiative.

There are many ways in which future work can be expanded for even greater impact. In light of the upcoming 2024 US elections, we are actively working to expand implementation of HDKs to more rural and community-based settings (including more FQHCs), expand participation of certain professional roles such as nurses, and achieve even greater distribution across states. Given the democratized, low-cost, low-input, and largely digital approach of our initiative, the main logistical hurdles to scaling even further are awareness and institutional buy-in. We hope that in disseminating our work and demonstrating the potential positive impact, particularly as it relates to equity, these particular barriers may be lessened. Future data collection should include additional demographic characteristics of participants, follow-up voting behaviors (e.g., will new voters from the 2020 initiative turn out in 2024?), and the relative yield of different engagement approaches (e.g., contact during emergency department versus primary care visits, QR codes on after-visit paperwork versus waiting room posters). Additionally, it would be meaningful to evaluate the perceptions and attitudes of both patients and HCWs about promotion of civic health. Furthermore, the US is not the only country in which marginalized groups experience disparities in both voting access and health outcomes, and our blueprint could be potentially adapted to international settings.

Finally, while our initiative’s grassroots toolkit approach amongst HCWs and partner institutions held great value in rapid expansion at the point-of-care, there are other avenues by which access to voting could be expanded on a larger systematic scale. National health agencies (e.g., Health Resources and Services Administration, Indian Health Services) could implement a top-down approach with standardized materials and workflows for healthcare settings. Automatic voter registration could occur alongside the signup process for health insurance (e.g., Medicaid, healthcare.gov), allowing for upwards of 1.2 million yearly voter registrations [40]. Public health departments – which are already value-aligned, primed for action, and frequently interface with the populations most affected by voting and health inequities – could implement concerted civic health promotion campaigns across the nation’s 3,006 counties. Efforts to improve voting access, literacy, and participation should be hand in hand with existing public health initiatives to improve health access, literacy, and participation in our most underserved and disenfranchised communities. Public health includes civic health, and the roots of equity in health and democracy are deeply intertwined.

## Figures and Tables

**Figure:**
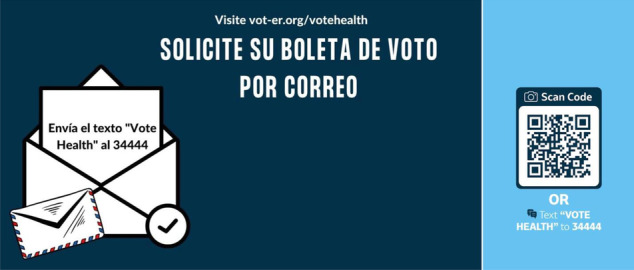
**Supplemental Figure 1.** Examples of digital HDK components, including a video teleconferencing background image in Spanish (left) and a smartphone screensaver with a QR code (right).

**Figure:**
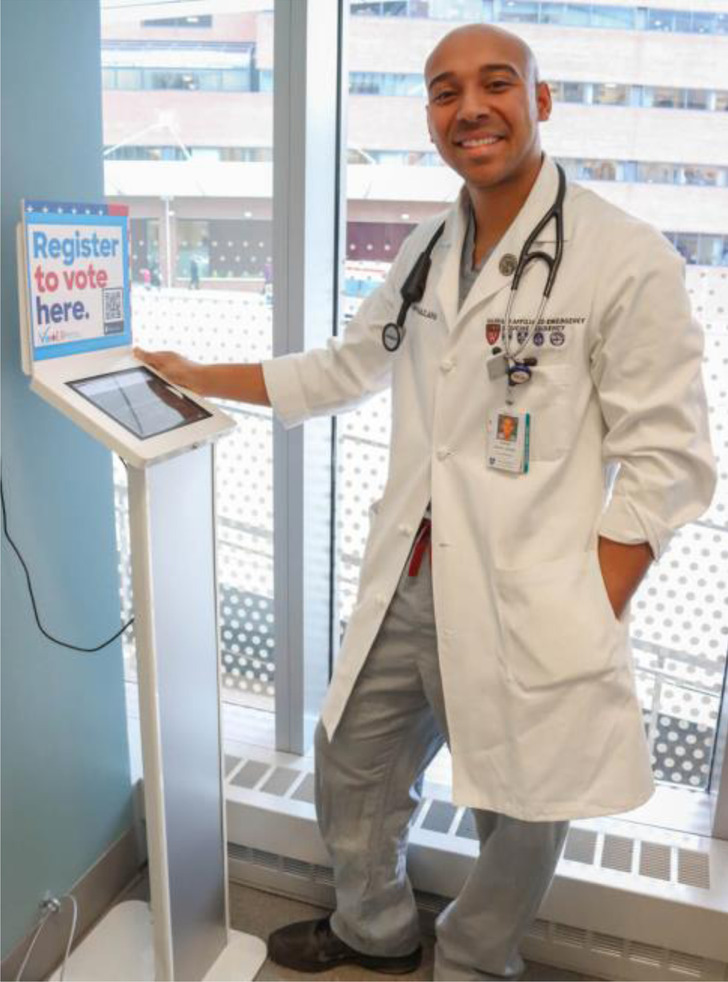
**Supplemental Figure 2.** Example of a Vot-ER kiosk used for voter registration in an emergency department waiting room, with demonstration by Dr. Alister Martin [32].

## Data Availability

Data from Vot-ER users are not publicly available in order to maintain anonymity of participants. All linked datasets used in this study are publicly available and appropriately referenced.

## Appendix 2

### Professional roles of individual HDK users

**Supplemental Table 1:** Summary of self-reported professional roles of HCWs who ordered individual HDKs

**Role**	**Number**	**Percentage**
Physician	7,554	57%
Attending	4,590	35%
Resident	1,987	15%
Fellow	246	1.9%
Not further specified	731	5.5%
Student	2,332	18%
Medical	2,209	17%
Social Work	31	0.23%
Nursing	26	0.20%
Physician Assistant	21	0.16%
Dental	6	0.05%
Occupational Therapy	3	0.02%
Physical Therapy	1	0.01%
Pharmacy	3	0.02%
Undergraduate	4	0.03%
Other Graduate	2	0.02%
Other / Not further specified	26	0.20%
Nurse	983	7.5%
Social Worker	637	4.8%
Physician Assistant	260	2.0%
Nurse Practitioner	212	1.6%
Hospital or Clinic Leadership	145	1.1%
Administrative or Support Staff	131	0.99%
Pharmacist or Pharmacy Assistant	95	0.72%
Medical or Nursing Assistant	77	0.58%
Technologist	59	0.45%
Physical Therapist	55	0.42%
Mental Health Professional	45	0.34%
Professor or Research Staff	40	0.30%
Occupational Therapist	31	0.23%
Paramedic or Emergency Medical Technician	24	0.18%
Other Educator	24	0.18%
Dietitian	22	0.17%
Midwife	17	0.13%
Vot-ER Staff	14	0.11%
Scribe	14	0.11%
Speech Language Pathologist	12	0.09%
Dentist or Dental Assistant	10	0.08%
Chaplain	10	0.08%
Trainer	9	0.07%
Advocate	9	0.07%
Volunteer	7	0.05%
Respiratory Therapist	6	0.05%
Case Manager	6	0.05%
Laboratory or Microbiology	5	0.04%
Child Life Specialist	5	0.04%
Librarian	4	0.03%
Community Health Worker	4	0.03%
Medical Interpreter	3	0.02%
Genetic Counselor	3	0.02%
Other^a^	48	0.36%
Not Answered	280	2.1%

^a^Additional roles with 2 or fewer persons (e.g., acupuncturist, music therapist, contact tracer)
